# Sleep practices and risk of stillbirth: implications for prevention and research

**DOI:** 10.1186/1471-2393-12-S1-A13

**Published:** 2012-08-28

**Authors:** Edwin A Mitchell

**Affiliations:** 1 University of Auckland, New Zealand

## 

Stillbirth research is hindered by a lack of a uniform definition and a lack of an internationally agreed protocol for the investigation of a death. However, the major issue is that not all stillbirths have an autopsy [1], and many are not investigated at all.

Mortality reviews (case series) are descriptive and hypothesis generating. In New Zealand the Perinatal and Maternal Mortality Review Committee reviews all deaths from 20 weeks gestation to 28 completed days after birth, or weighing at least 400g if gestation is unknown [2]. In 2009 the stillbirth rate was 6.3/1000 births. 25% were unexplained, and half of these occurred at term. 22% were not investigated. Maori and Pacific mothers were at higher risk and rates were increased in the most deprived socioeconomic quintile. Teenage mothers and those 40+ years were also at higher risk. Maternal obesity, multiple pregnancy and maternal medical conditions were also associated with risk of stillbirth. Only 15% of stillbirths were potentially avoidable. Although much can be learnt from examining national mortality databases, these do not allow examination of risk factors which are operating close to the demise of the fetus.

Case control studies are an efficient way to examine such risk factors. A small number of studies have been completed [3,4] or are still collecting data. The Auckland Stillbirth Study, a three year case-control study, had a particular focus on modifiable risk factors, including that related to maternal sleep [3]. We found an increased risk of stillbirth with non-left sleep position, regular sleep during day-time, more than 8 hours of night-time sleep and getting up to the toilet once or less per night [5]. The prevalence of non left sided sleep position in this study was 57.3% and the population attributable risk for non-left sided sleep position was 37%. Therefore, if there is a causative pathway between maternal sleep position and late stillbirth, over a third of late stillbirths (in high income countries) could be attributable to maternal sleep position. Further studies need to be conducted as soon as possible to determine whether this finding is reproducible and if confirmed, it would then be appropriate to instigate public health campaigns to encourage women to go to sleep on their left side in late pregnancy.

Research is needed to show how best to implement the findings. One testable hypothesis is that sleeping on the left side of the bed encourages mothers to sleep on their left side. A survey in Auckland is currently underway to examine this.

Furthermore, physiological studies of the pregnant mother and the fetus are required, such as examining the effect of maternal body position on cardiac output, uterine blood flow and on fetal wellbeing. There needs to be sleep studies of normal pregnant mothers in late pregnancy as well as longitudinal studies over the last trimester and possibly beginning at 20 weeks gestation.
